# Fluoxetine Ameliorates Atopic Dermatitis-Like Skin Lesions in BALB/c Mice through Reducing Psychological Stress and Inflammatory Response

**DOI:** 10.3389/fphar.2016.00318

**Published:** 2016-09-13

**Authors:** Yanxi Li, Long Chen, Yehong Du, Daochao Huang, Huili Han, Zhifang Dong

**Affiliations:** ^1^Chongqing Key Laboratory of Translational Medical Research in Cognitive Development and Learning and Memory Disorders, Children’s Hospital of Chongqing Medical UniversityChongqing, China; ^2^Ministry of Education Key Laboratory of Child Development and Disorders, Children’s Hospital of Chongqing Medical UniversityChongqing, China; ^3^The Chongqing Hospital of Traditional Chinese MedicineChongqing, China

**Keywords:** atopic dermatitis, fluoxetine, anti-inflammation, psychological stress, itching

## Abstract

Atopic dermatitis (AD) is a common chronic inflammatory skin disorder, and patients with AD suffer from severe psychological stress, which markedly increases the prevalence rate of depression and anxiety disorders in later life. Fluoxetine, a selective serotonin reuptake inhibitor, has recently been reported to exert anti-inflammatory and immunosuppressive effects. However, it is unclear whether fluoxetine is effective in the treatment of AD through reducing psychological stress and inflammatory reaction. Here, we reported that a BALB/c mouse model of AD was induced by application of 2,4-dinitrochlorobenzene (DNCB) onto hairless dorsal skin. Chronic fluoxetine treatment (10 mg/kg per day, i.p.) significantly attenuated AD-like symptoms, as reflected by a dramatic decrease in scratching bouts, as well as a decrease in anxiety- and depressive-like behaviors. Furthermore, these behavioral changes were accompanied by a significant decrease in epidermal thickness, the number of mast cells in skin tissue, mRNA levels of interleukin-4 (IL-4) and IL-13 in the spleen, as well as serum immunoglobulin E (IgE) in the DNCB-treated mice by treatment with fluoxetine. Taken together, these results indicate that fluoxetine may suppress psychological stress and inflammatory response during AD development, and subsequently ameliorate AD symptoms, suggesting that fluoxetine may be a potential therapeutic agent against AD in clinic.

## Introduction

Atopic dermatitis (AD), also known as atopic eczema, is a common chronic inflammatory skin disease that is characterized by intense itching and recurrent eczematous lesions. The prevalence rate of AD is rising dramatically, especially in developed countries, and it now affects 10–30% of children and 1–10% of adults (Leung et al., 2004; Montes-Torres et al., 2015; Weidinger and Novak, 2015), leading to a significant reduction in quality of life and economic burden (Berke et al., 2012). The pathogenesis of AD is not very clear, but it seems to be correlated with specific immune and inflammatory mechanisms. It has been reported that repeated applications of 2,4-dinitrochlorobenzene (DNCB) to mouse skin induced AD-like skin lesions, which is associated with a significant increase in serum immunoglobulin E (IgE) and T-helper (Th) 2 cytokines such as interleukin-4 (IL-4) and IL-13 at the chronic dermatitis site (Kitagaki et al., 1995; Harada et al., 2005). Further clinic studies have shown that these immunological changes are also observed in patients with AD (Leung et al., 2004; Liu et al., 2011). Therefore, most cases of AD currently are treated with emollients and topical anti-inflammatory agents such as topical corticosteroids and the topical calcineurin inhibitors. Many patients can be managed effectively, however, considerable number of patients still suffers from relapsing intolerable AD. In addition, these pharmacological therapies may cause various side effects including skin atrophy, telangiectases, purpura, and striae formation (Del Rosso and Friedlander, 2005). Thus, the use of less toxic alternative therapeutic agent against AD is imperative.

Meanwhile, a growing body of evidence has shown that patients with AD suffer from a severe psychological stress, which markedly increases the prevalence rate of depression and anxiety disorders (Wahlgren, 1992; Ginsburg et al., 1993; Hashiro and Okumura, 1997; Cheng et al., 2015), and in turn, stress may aggravate AD symptoms. Thus, reduction of depressive- and/or anxiety-related emotions by inhibiting psychological stress may be a potential method for the treatment of AD. Indeed, previous studies have shown that paroxetine, a selective serotonin reuptake inhibitor (SSRI), is effective in the treatment of AD-like lesions in NC/Nga mice and chronic pruritus in patients (Zylicz et al., 2003; Jiang et al., 2007; Stander et al., 2009). Our recent study has reported that another SSRI fluoxetine, which has been widely used as an antidepressant agent in clinic, displayed anti-stress effects (Han et al., 2015). Furthermore, fluoxetine has also shown anti-inflammatory and direct immunosuppressive effects such as suppression of T cell activation, cytokine secretion and proliferation and induction of apoptosis *in vitro* and *in vivo* (Janssen et al., 2010; Gobin et al., 2013, 2014). Thus, it is reasonable to propose that fluoxetine may ameliorate AD through reducing psychological stress and inflammatory response.

To test this hypothesis, we treated BALB/c mice with DNCB to induce human AD-like skin lesions, and assessed the inhibitory effect of fluoxetine on the development of dermatitis and to obtain basic information about the usefulness of fluoxetine in the treatment of AD.

## Materials and Methods

### Animals

Six-week-old male BALB/c mice were obtained from Chongqing Medical University Animal House Center and maintained at Children’s Hospital of Chongqing Medical University Animal Care Centre. Animals were housed in plastic cages in a temperature-controlled (21°C) colony room on a 12/12 h light/dark cycle. Food and water were available *ad libitum*. All experiments and procedures were approved by Chongqing Medical University Animal Care and Use Committee. All efforts were made to minimize the number of animals used.

### Drugs

Fluoxetine and DNCB were purchased from Sigma-Aldrich Co. (St. Louis, MO, USA). DNCB was dissolved in acetone-olive oil (4:1, v/v) and fluoxetine was dissolved in 0.9% sterile saline at a concentration of 10 mg/ml. The drug application and experimental tests were performed in a double-blinded manner.

### Induction of AD-Like Skin Lesions and Fluoxetine Treatment

**FIGURE 1:**
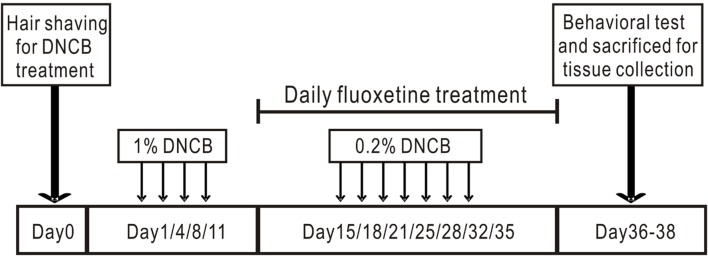
**Experimental protocol for induction of atopic dermatitis in mouse model.** The back skin of mice was applied repeatedly with DNCB for 5 weeks to induce AD. Fluoxetine was intraperitoneally administrated from the third week together with DNCB.

Induction of AD-like skin lesions procedure is described in **Figure 1** as described previously (Kim S. R. et al., 2014). The backs of mice were shaved with an electric clipper and depilatory cream a day before DNCB sensitization. The DNCB sensitization and challenge were performed for 5 weeks. For the sensitization process, a 1 cm × 1 cm gauze-attached patch was applied with 200 μl of 1% DNCB and attached to the shaved area twice per week. Two weeks after sensitization, the back skin was challenged with 200 μl of a 0.2% DNCB solution twice per week. This procedure was repeated for 3 weeks and fluoxetine was intraperitoneally administrated together. Behavioral tests were performed within 3 days after DNCB and fluoxetine treatments. At the end of the experiment, mice were sacrificed and samples were collected to evaluate the effects of DNCB and fluoxetine treatments (**Figure 1**).

### Clinical Scoring of Skin Lesions

The severity of DNCB-induced skin lesions was clinically assessed as previously described (Matsuda et al., 1997; Kim et al., 2008). Briefly, the total clinical severity score for skin lesions was calculated as the sum of the individual scores (0, no symptom; 1, mild; 2, moderate; 3, severe) for the following four AD signs and symptoms: erythema/hemorrhage, edema, excoriation/erosion, and scaling/dryness.

### Scratching Behavior

The animals were placed into an observation chamber for 10 min for acclimatization before the measurement of scratching behavior. The scratching behavior was defined as the hind limb scratches directed to the shaved area. The number of scratching behaviors was counted for 30 min each time.

### Elevated Plus Maze

Mice were placed in the center of a plus maze (each arm 30 cm) that was elevated 50 cm above the floor with two opposite open arms and two opposite closed arms (10-cm-tall walls on the closed arms) arranged at right angles. The number of entries and time spent in the closed and open arms were monitored for 10 min by ANY-Maze Video Tracking System. The maze was cleaned with 70% ethanol and water between tests.

### Forced Swimming

Mice were placed in a cylinder of water (temperature 24–25°C; 10 cm in diameter, 30 cm in height) for 10 min. The depth of water was set to prevent animals from touching the bottom with their hind limbs. The latency to immobility, which was defined as floating or the least movement to maintain the head above the water, was recorded by ANY-Maze Video Tracking System (Stoelting Co.) from the side.

### Histological Analysis

A portion of the skin biopsies were fixed in 4% paraformaldehyde. The skin sections (4 μm thick) were stained with hematoxylin and eosin (H&E) to evaluate the epidermal hyperplasia and the other sections were stained with toluidine blue to evaluate the infiltration of mast cells. Mast cells were counted in five parts of high-power fields (HPF) at 400× magnification.

### Measurement of Serum IgE

Blood samples were collected by cardiac puncture under anesthesia, and sera were collected by centrifugation and stored at -80°C until use. Total IgE levels in plasma were determined by sandwich ELISA using the Sigma mouse IgE ELISA kit (St. Louis, MO, USA).

### Cytokine Analysis by Real-Time PCR

Total RNA was extracted from spleen tissue with the TRIzol reagent (Takara, Otsu, Shiga, Japan) according to the manufac turer’s instruction. The quantity and purity of total RNA were determined with a Nanodrop reader (Nanodrop Technologies, Wilmington, DE, USA). One microgram of total RNA was converted to the first-strand DNA with PrimeScript RT reagent Kit with gDNA Eraser (Takara, Otsu, Shiga, Japan) reverse transcriptase (Takara, Otsu, Shiga, Japan) and RNAsin (Takara, Otsu, Shiga, Japan). cDNA was amplified using gene-specific primers and SYBR^®^ Premix Ex Taq^TM^ II (Takara, Otsu, Shiga, Japan). Primer sequences were as follows: Interferon-γ (IFN-γ) (forward: 5′-CTCAAGTGGCATAGATGT-3′, reverse: 5′-GAGATAATCTGGCTCTGCAGGATT-3′); IL-2 (forward: 5′-CTCTACAGCGGAAGCACAGCA-3′, reverse: 5′-TGCCGCAGAGGTCCAAGTT-3′); IL-4 (forward: 5′-TGAACGAGGTCACAGGAGAAGG-3′, reverse: 5′-CACCTTGGAAGCCCTACAGACA-3′); IL-13 (forward: 5′-AGCATGGTATGGAGTGTGGACCTG-3′, reverse: 5′-CAGTTGCTTTGTGTAGCTGAGCAG-3′); β-actin (forward: 5′-GCACCACACCTTCTACAATGAGC-3′, reverse: 5′-GGATAGCAGCCTGGATAGCAAC-3′). The relative expression levels of target genes were normalized using β-actin as an internal control.

### Statistical Analyses

All data are expressed as the mean ± SEM. Statistical analysis of the results were performed by one-way analysis of variance (ANOVA) followed by Turkey’s test. Significance level was set at *p* < 0.05.

## Results

### Chronic Fluoxetine Treatment Alleviates DNCB-Induced AD Symptoms

**FIGURE 2:**
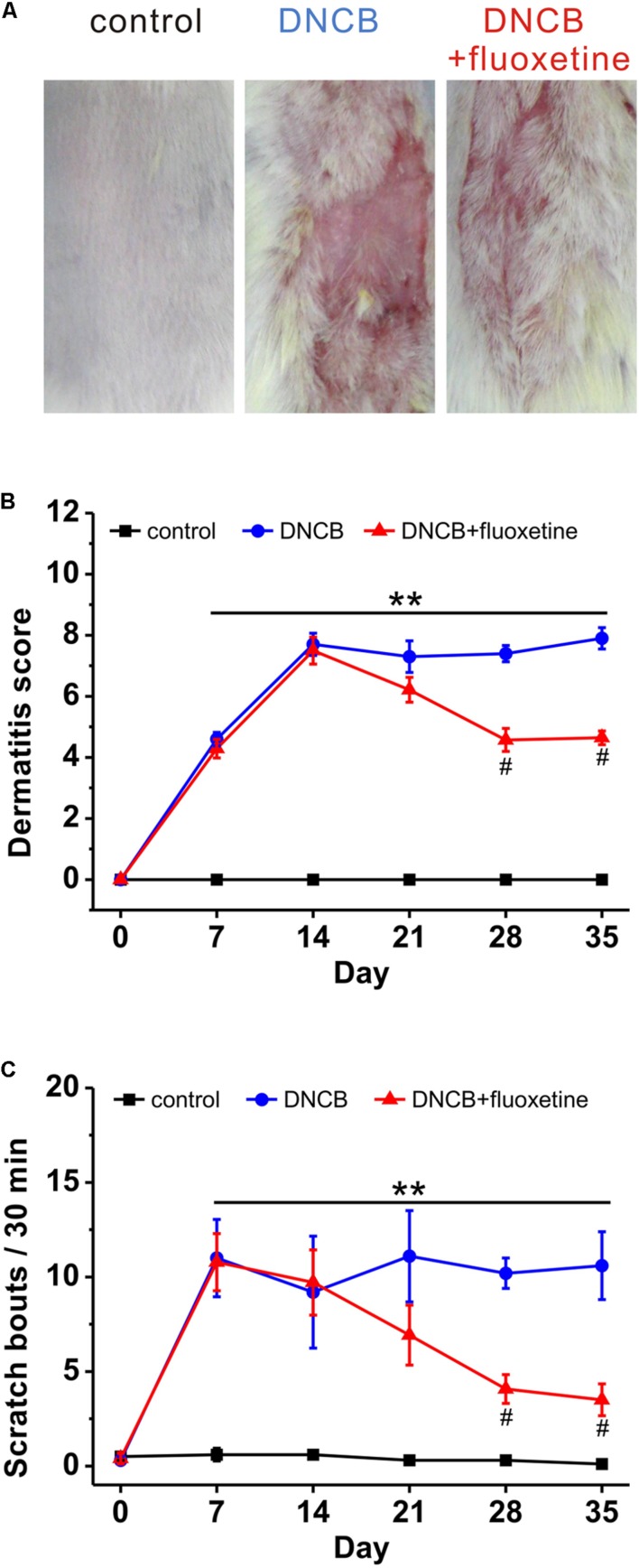
**The preventive effect of chronic fluoxetine treatment on AD-like skin lesions induced by DNCB treatment.** **(A)** Photographs showing skin lesions in the different groups of experimental mice. **(B)** The severity of clinical symptoms of the skin lesions was evaluated macroscopically and calculated as the sum of the individual scores once a week for the following four AD signs and symptoms: erythema/hemorrhage, edema, excoriation/erosion and scaling/dryness. **(C)** The frequency of scratching behavior was observed and calculated once a week in the different treatment group. *n* = 10 for control and DNCB, *n* = 14 for DNCB+fluoxetine. ^∗∗^*P* < 0.01 compared with control, and ^#^*p* < 0.05 compared with DNCB group.

Previous studies have shown that paroxetine, a selective SSRI, is effective in the treatment of AD in both animal model and patients, with no apparent side-effects (Zylicz et al., 2003; Jiang et al., 2007; Stander et al., 2009). To determine whether fluoxetine is effective in the treatment of AD-like skin lesions, we chronically treated AD mice with fluoxetine (10 mg/kg, i.p.; **Figure 1**). Consistent with previous reports (Kim H. et al., 2014; Kim S. R. et al., 2014; Yoon et al., 2015), AD-like lesions developed following repeated application of DNCB to the back skin (**Figure 2A**). More importantly, chronic fluoxetine administration dramatically alleviated skin lesions (**Figure 2A**). To further compare the skin lesions of the mice with those of human AD (Mihm et al., 1976; Piloto Valdes et al., 1990), clinical severity of the dermatitis was scored as previously described (Leung et al., 1990). The result showed that DNCB treatment significantly increased the skin lesion score compared with normal control, whereas the skin lesion score was significantly decreased after fluoxetine treatment (control: *n* = 10; DNCB: *n* = 10; DNCB+fluoxetine: *n* = 14; **Figure 2B**). In addition, chronic fluoxetine treatment significantly suppressed DNCB-induced scratching behaviors (control: *n* = 10; DNCB: *n* = 10; DNCB+fluoxetine: *n* = 14; **Figure 2C**). Taken together, these results suggest that chronic fluoxetine treatment alleviates the DNCB-induced skin lesions.

### Chronic Fluoxetine Treatment Reduces Depressive- and Anxiety-Like Behaviors in AD Mice

**FIGURE 3:**
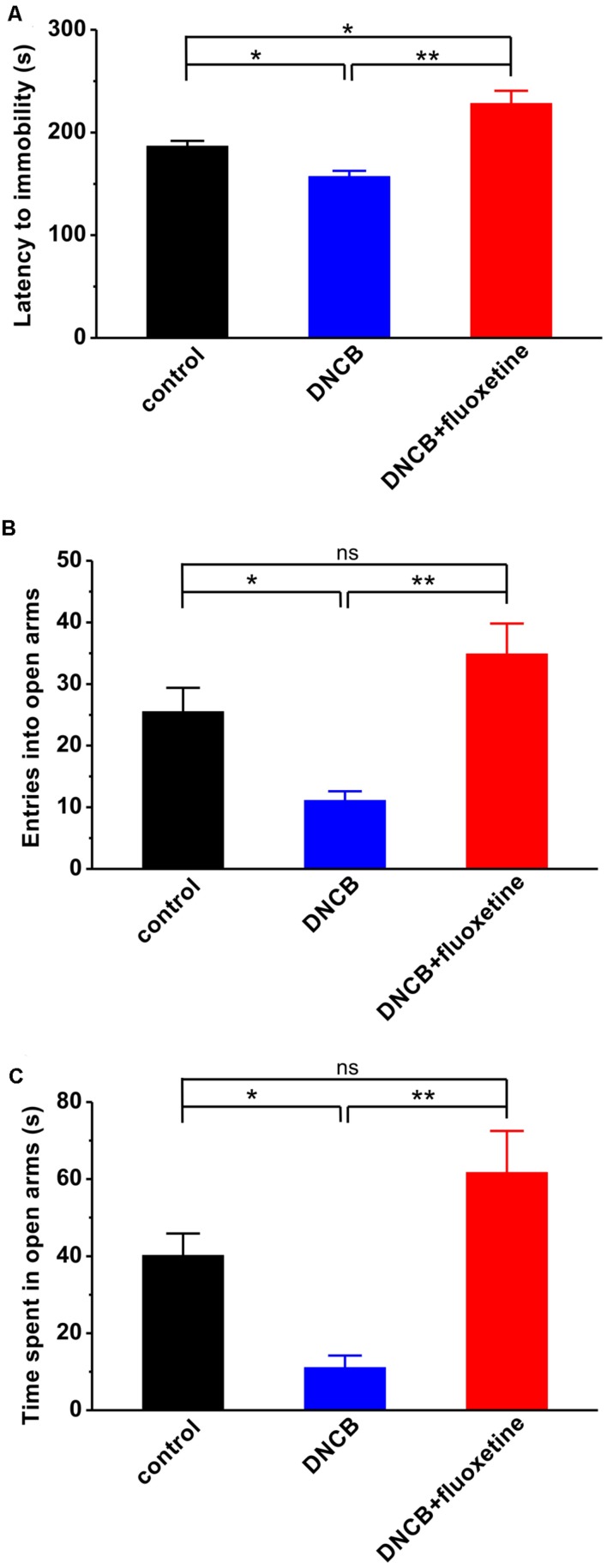
**Effects of chronic fluoxetine treatment on depressive- and anxiety-like behaviors induced by DNCB treatment.** **(A)** The latency to immobility was measured during forced swimming test in the different groups of experimental mice. Both the number of entries into open arms **(B)** and the time spent in open arms **(C)** were measured during elevated plus maze test. *n* = 10 for control and DNCB, *n* = 14 for DNCB+fluoxetine. ^∗^*P* < 0.05, ^∗∗^*P* < 0.01.

A growing body of evidence has shown that AD in both adolescence and adulthood increases the risk of developing major depression and anxiety disorders in later life (Wahlgren, 1992; Ginsburg et al., 1993; Hashiro and Okumura, 1997; Cheng et al., 2015), and fluoxetine has been widely used as an antidepressant agent in clinic. Thus, we next introduced different behavioral tests, including forced swimming and elevated plus maze, to determine whether the alleviating effect of fluoxetine on AD is associated with its antipsychotic effect. Compared to normal controls, AD mice displayed apparent depressive-like symptom, as reflected by much shorter latency to immobility during forced swimming test (control: *n* = 10, 186.8 ± 5.0 s; DNCB: *n* = 10, 157.2 ± 10.4 s, *p* < 0.05 vs. control; **Figure 3A**). In agreement with an anti-depressive effect of fluoxetine, it succeeded in abolishing the influence of DNCB on depressive behavior (DNCB+fluoxetine: *n* = 14, 228.3 ± 15.2 s, *p* < 0.05 vs. control, *p* < 0.01 vs. DNCB; **Figure 3A**).

Next, using elevated plus maze paradigm, we tested the effect of fluoxetine on anxiety-like behavior in AD. The results showed that both the number of entry into open arms (control: *n* = 10, 25.5 ± 3.9; DNCB: *n* = 10, 11.1 ± 1.5, *p* < 0.05 vs. control; **Figure 3B**) and the time spent in open arms (control: *n* = 10, 40.2 ± 5.5 s; DNCB: *n* = 10, 11.1 ± 3.1 s, *p* < 0.05 vs. control; **Figure 3C**) were significantly reduced in DNCB-treated mice during elevated plus maze test. Similar to forced swimming test, chronic fluoxetine treatment was able to restore the number of entry into the open arms (DNCB+fluoxetine: *n* = 14, 34.9 ± 4.9, *p* < 0.05 vs. control, *p* < 0.01 vs. DNCB; **Figure 3B**) and the time in open arms (DNCB+fluoxetine: *n* = 14, 61.7 ± 10.8 s, *p* < 0.05 vs. control, *p* < 0.01 vs. DNCB; **Figure 3C**) to control levels, indicating chronic fluoxetine administration reduces anxiety during AD development.

### Chronic Fluoxetine Treatment Suppresses Tissue Inflammation and the Accumulation of Mast Cells in AD-Like Skin Lesions

**FIGURE 4:**
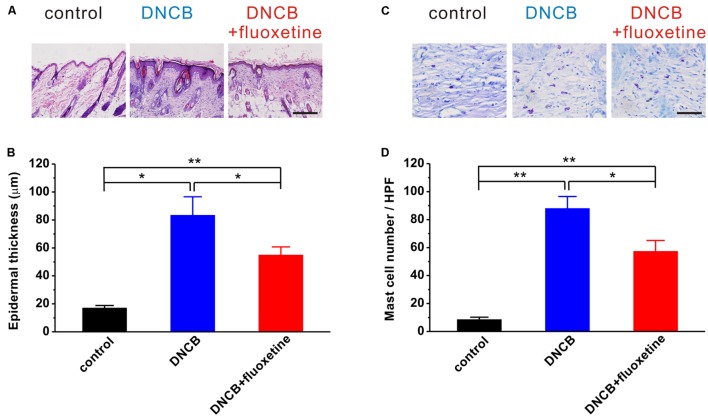
**Histopathological findings showing the preventive effect of chronic fluoxetine treatment on DNCB-induced AD-like skin lesions.** **(A)** Skin sections (4 μm thick) were stained with H&E. Scale bar is 100 μm. **(B)** Epidermal thickness was analyzed in the H&E-stained sections. **(C)** Skin sections were stained with toluidine blue. Scale bar is 50 μm. **(D)** The number of mast cells were counted in five high-power fields (HPF) chosen at random in each toluidine blue-stained slide by three different pathologists. *n* = 10 for control and DNCB, *n* = 14 for DNCB+fluoxetine. ^∗^*P* < 0.05, ^∗∗^*P* < 0.01.

It has been widely accepted that AD is a common chronic inflammatory skin disease. We next wanted to determine the alterations of tissue inflammation and the accumulation of mast cells in AD-like skin lesions. Consistent with previous reports (Kim S. R. et al., 2014; Yoon et al., 2015), histopathological examination showed that the skins of the DNCB-treated group showed markedly epidermal hyperplasia as compared to the normal group, whereas this hyperplasia was significantly reduced by chronic fluoxetine treatment (control: *n* = 10, 17.0 ± 1.8 μm; DNCB: *n* = 10, 83.4 ± 13.2 μm, *p* < 0.01 vs. control; DNCB+fluoxetine: *n* = 14, 54.9 ± 5.8 μm, *p* < 0.01 vs. control, *p* < 0.05 vs. DNCB; **Figures 4A,B**). Similarly, the number of mast cells in the dermis of DNCB-treated mice was markedly increased as compared to the normal group, whereas this increment was significantly suppressed by chronic fluoxetine treatment (control: *n* = 10, 8.5 ± 1.7; DNCB: *n* = 10, 88.0 ± 8.5, *p* < 0.01 vs. control; DNCB+fluoxetine: *n* = 14, 57.3 ± 7.8, *p* < 0.01 vs. control, *p* < 0.05 vs. DNCB; **Figures 4C,D**).

### Chronic Fluoxetine Treatment Inhibits DNCB-Induced Serum IgE Elevation

**FIGURE 5:**
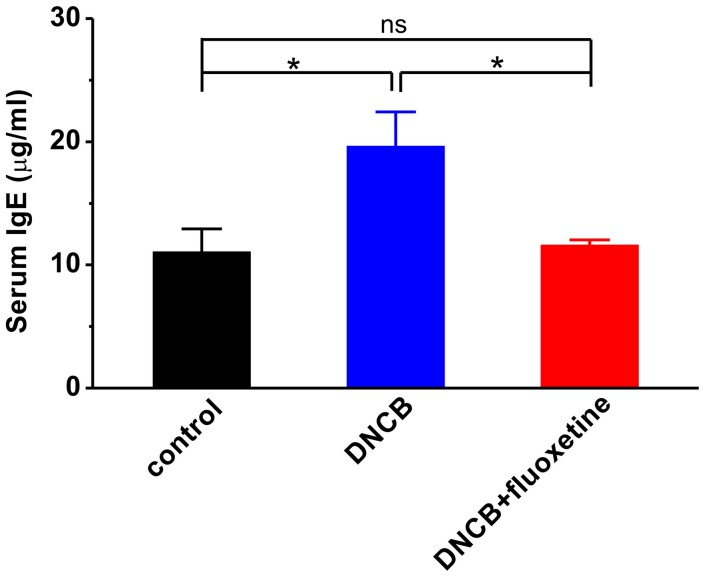
**Effects of chronic fluoxetine treatment on serum levels of lgE in DNCB-treated mice.** Total serum levels of total IgE were determined by ELISA. *n* = 4–6 in each group. ^∗^*P* < 0.05.

Since serum IgE level is correlated with the severity of AD (Arshad and Holgate, 2001; Belloni et al., 2008; Ibler and Jemec, 2015; Montes-Torres et al., 2015), and the level of IgE is associated with Th2 or Th1 immunity (Snapper and Paul, 1987), we examined the serum level of IgE to evaluate the effect of fluoxetine on systemic Th1 and Th2 immunities in DNCB-treated mice. As shown in **Figure 5**, the serum level of IgE was markedly increased in the DNCB-treated group compared to normal control (control: *n* = 4, 11.1 ± 1.9 μg/ml; DNCB: *n* = 6, 19.6 ± 2.8 μg/ml, *p* < 0.01 vs. control; **Figure 5**). Chronic fluoxetine treatment restored the serum IgE to normal control level (DNCB+fluoxetine: *n* = 5, 11.6 ± 0.4 μg/ml, *p* > 0.05 vs. control, *p* < 0.05 vs. DNCB; **Figure 5**).

### Chronic Fluoxetine Treatment Reduces Spleen Cytokines in AD Mice

**FIGURE 6:**
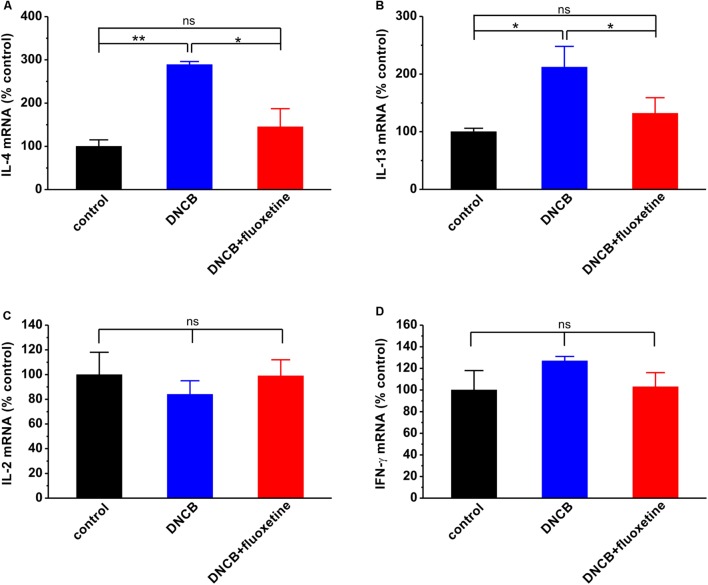
**Effects of chronic fluoxetine treatment on mRNA expressions of cytokines.** IL-4 **(A)**, IL-13 **(B)**, IL-2 **(C)** and IFN-γ **(D)** mRNA expressions were determined by real-time PCR. *n* = 4–6 in each group. ^∗^*P* < 0.05, ^∗∗^*P* < 0.01.

In the past decades, SSRIs including fluoxetine have been shown to exert several immunological effects, such as alteration of cytokine secretion (Janssen et al., 2010; Gobin et al., 2013, 2014). To evaluate the effects of fluoxetine on Th1 and Th2 immunities in AD, we examined the expression of IL-2, IFN-γ, IL-4, and IL-13 mRNAs in the spleen of experimental mice. The results showed that the IL-4 (control: *n* = 4, 100.0 ± 14.7; DNCB: *n* = 4, 288.6 ± 7.1, *p* < 0.01 vs. control; **Figure 6A**) and IL-13 (control: *n* = 6, 100.0 ± 6.5; DNCB: *n* = 5, 212.5 ± 36.1, *p* < 0.05 vs. control; **Figure 6B**) mRNA expressions were dramatically increased, whereas the IL-2 (control: *n* = 6, 100.0 ± 18.0; DNCB: *n* = 6, 84.0 ± 11.5, *p* > 0.05 vs. control; DNCB+fluoxetine: *n* = 5, 99.1 ± 12.6, *p* > 0.05 control, *p* > 0.05 vs. DNCB; **Figure 6C**) and IFN-γ (control: *n* = 6, 100.0 ± 17.6; DNCB: *n* = 6, 126.8 ± 4.4, *p* > 0.05 vs. control; DNCB+fluoxetine: *n* = 6, 102.6 ± 12.9, *p* > 0.05 control, *p* > 0.05 vs. DNCB; **Figure 6D**) mRNA expressions remained unchanged in DNCB-treated mice as compared to the normal group. More importantly, chronic fluoxetine treatment suppressed the DNCB-induced increase of IL-4 (DNCB+fluoxetine: *n* = 5, 145.2 ± 42.3, *p* > 0.05 control, *p* < 0.05 vs. DNCB; **Figure 6A**) and IL-13 mRNA expressions (DNCB+fluoxetine: *n* = 6, 132.1 ± 27.3, *p* > 0.05 control, *p* < 0.05 vs. DNCB; **Figure 6B**)

## Discussion

In the present study, we confirm that repeated DNCB treatment induces the AD-like pathology, and demonstrate that chronic fluoxetine treatment reduces DNCB-induced inflammatory response and subsequently ameliorate AD symptoms in BALB/c mice. Therefore, our current results indicate that fluoxetine may effectively prevent AD symptoms.

It has been reported that SSRI such as paroxetine can inhibit the development of AD-like lesions in NC/Nga mice and alleviate chronic pruritus in patients (Zylicz et al., 2003; Jiang et al., 2007; Stander et al., 2009). However, the underlying mechanism of antipruritic effect of SSRI remains unclear at present. It is well documented that AD is an inflammatory skin disease characterized by pruritic and eczematous skin lesions, extensive inflammatory cell infiltration and release of pro-inflammatory mediators (Morar et al., 2006). Previous study has shown that IgE expression causes both acute and chronic phase skin inflammations, suggesting that the upregulation of total serum IgE may be a hallmark of AD (Arshad and Holgate, 2001). Further study has shown that the level of IgE is associated with Th2 or Th1 immunity, in turn, Th2 has powerful effect in stimulating the expression of IgE (Snapper and Paul, 1987). Therefore, although the mechanism for the anti-AD action of fluoxetine remains unclear, the downregulation of serum IgE and Th2 immunity may be a principal mechanism for the fluoxetine action. Indeed, in the present study, we found that chronic fluoxetine treatment reduced total IgE level and suppressed Th2 such as IL-4 and IL-13 mRNA expressions in DNCB-treated mice. This hypothesis is further supported by more recent reports. For example, SSRIs including fluoxetine have been shown to exert several immunosuppressive effects, such as decreased lymphocyte proliferation and reduced pro-inflammatory cytokine secretion (Janssen et al., 2010; Gobin et al., 2013, 2014). On the other hand, fluoxetine significantly decreases lymphocyte proliferation, which is resulted from elevated central serotonin (5-HT) neurotransmission and activation of central 5-HT2 receptors (Pellegrino and Bayer, 2002). In addition, fluoxetine also can exert immunosuppressive effect via suppressing intra-cellular Ca^2+^ signaling in Jurkat T lymphocytes through depletion of Ca^2+^ from intracellular stores (Gobin et al., 2015).

Alternatively, fluoxetine may modulate psycho-emotion and subsequently prevent AD symptoms. Previous study has shown that the majority of patients with AD are subjected to psychological stress, and in turn, stress aggravates their pruritus, suggesting that pruritus might be closely related with emotional distress in AD (Wahlgren, 1992). Fluoxetine is frequently used in clinic to treat neuropsychiatric disorders, such as major depressive disorder, obsessive-compulsive disorder, post-traumatic stress disorder, and anxiety disorder (McIntyre and Katzman, 2003; Pinna et al., 2009). Thus, it is reasonable to assume that fluoxetine may play anti-AD action through the inhibition of psychological stress. Indeed, in the present study, we found that chronic fluoxetine treatment led to a significant decrease in DNCB-induced depressive- and anxiety-like behaviors (**Figure 3**). Notably, chronic treatment with fluoxetine impairs adult neurogenesis and locomotor activity in mice (Ohira and Miyakawa, 2011; Zheng et al., 2011). Thus, future experiments determining the potential side effects of chronic treatment with fluoxetine will be helpful to fluoxetine in the treatment of AD.

In summary, we demonstrate that chronic fluoxetine treatment is an effective means to reduce DNCB-induced scratching bouts, depressive/anxiety-like behaviors, skin lesions and serum IgE levels in mice model of AD, and these effects are mediated by regulating pro-inflammatory cytokines, such as IL-4 and IL-13. These results suggest that fluoxetine may be a potential therapeutic agent against AD in clinic; nevertheless, further investigations would be necessary to clarify its molecular mechanisms of action in AD.

## Author Contributions

YL, LC, YD, and DH performed the research. HH and ZD designed the research study. YL, HH, and ZD contributed essential reagents or tools. YL, LC, YD, and ZD analyzed the data. HH and ZD wrote the manuscript.

## Conflict of Interest Statement

The authors declare that the research was conducted in the absence of any commercial or financial relationships that could be construed as a potential conflict of interest.
